# Social visual attentional engagement and memory in Phelan-McDermid syndrome and autism spectrum disorder: a pilot eye tracking study

**DOI:** 10.1186/s11689-021-09400-2

**Published:** 2021-12-04

**Authors:** Sylvia B. Guillory, Victoria Z. Baskett, Hannah E. Grosman, Christopher S. McLaughlin, Emily L. Isenstein, Emma Wilkinson, Jordana Weissman, Bari Britvan, M. Pilar Trelles, Danielle B. Halpern, Joseph D. Buxbaum, Paige M. Siper, A. Ting Wang, Alexander Kolevzon, Jennifer H. Foss-Feig

**Affiliations:** 1 Seaver Autism Center for Research and Treatment, Icahn School of Medicine at Mount Sinai, New York, USA; 2 Department of Psychiatry, Icahn School of Medicine at Mount Sinai, 1 Gustave L. Levy Place, Box 1230, New York, NY 10029 USA; 3 School of General Studies, Columbia University, New York, USA; 4 Department of Medicine, University of Rochester, Rochester, USA; 5 Mindich Child Health and Development Institute, Icahn School of Medicine at Mount Sinai, New York, USA; 6 Department of Neuroscience, Icahn School of Medicine at Mount Sinai, New York, USA; 7 Friedman Brain Institute, Icahn School of Medicine at Mount Sinai, New York, USA; 8 Department of Genetics and Genomic Sciences, Icahn School of Medicine at Mount Sinai, New York, USA; 9 Department of Pediatrics, Icahn School of Medicine at Mount Sinai, New York, USA

**Keywords:** Visual attention, Autism spectrum disorder, Recognition memory, Phelan-McDermid syndrome, Social processing, Eye tracking

## Abstract

**Background:**

The current study used eye tracking to investigate attention and recognition memory in Phelan-McDermid syndrome (PMS), a rare genetic disorder characterized by intellectual disability, motor delays, and a high likelihood of comorbid autism spectrum disorder (ASD). Social deficits represent a core feature of ASD, including decreased propensity to orient to or show preference for social stimuli.

**Methods:**

We used a visual paired-comparison task with both social and non-social images, assessing looking behavior to a novel image versus a previously viewed familiar image to characterize social attention and recognition memory in PMS (*n* = 22), idiopathic ASD (iASD, *n* = 38), and typically developing (TD) controls (*n* = 26). The idiopathic ASD cohort was divided into subgroups with intellectual disabilities (ID; developmental quotient < 70) and without (developmental quotient > 70) and the PMS group into those with and without a co-morbid ASD diagnosis.

**Results:**

On measures of attention, the PMS group with a comorbid ASD diagnosis spent less time viewing the social images compared to non-social images; the rate of looking back and forth between images was lowest in the iASD with ID group. Furthermore, while all groups demonstrated intact recognition memory when novel non-social stimuli were initially presented (pre-switch), participants with PMS showed no preference during the post-switch memory presentation. In iASD, the group without ID, but not the group with ID, showed a novelty preference for social stimuli. Across indices, individuals with PMS and ASD performed more similarly to PMS without ASD and less similarly to the iASD group.

**Conclusion:**

These findings demonstrate further evidence of differences in attention and memory for social stimuli in ASD and provide contrasts between iASD and PMS.

## Background

Phelan-McDermid syndrome (PMS) is a rare developmental disorder that is characterized by broad cognitive delays, delayed or absent speech, and hypotonia [35, 38]. Caused by a deletion or mutation in the chromosome region 22q13.3, disruption of the *SHANK3* gene causes the behavioral phenotype of the disorder [16, 22], with larger deletion sizes correlating with increased symptom severity [46, 48]. In animal models, deficient *Shank3* expression results in atypical cognitive processes that include impaired visual discrimination and memory [14, 25] and diminished expressive communication [4, 6, 55]), yet intact social approach behaviors [14, 17]. Many of these features are also seen in the clinical phenotype. Indeed, individuals with PMS show persistent communication deficits [34, 48, 60]. A majority have minimal or absent speech [37, 39], with intellectual disabilities (ID) reported in most cases [16, 48]. Additional behavioral characteristics include sleep disturbances, impulsivity, and inattention [8, 16, 27, 40]. In a subset of individual with PMS due to point mutations, De Rubeis et al. [16] reported that up to 65% are hyperactive, with broad attentional problems.

Studies have reported a high prevalence of autism spectrum disorder (ASD) diagnoses among individuals with PMS [31, 48]; in fact, *SHANK3* haploinsufficiency is among the most common single-gene causes of ASD and ID [5, 28]. ASD is characterized by social and communication deficits as well as restricted interests and repetitive behaviors [2]. Though ASD is itself heterogeneous, within the social domain, these impairments can include poorly modulated eye contact, diminished awareness and understanding of the thoughts and intention of others, and difficulties in social engagement [50]. Specific to ASD symptoms, PMS individuals express deficits in social communication that involve social reciprocity, engagement, and play skills; up to 85% meet criteria for ASD [48]. However, in comparison to typical patterns within idiopathic ASD (iASD), a lower proportion of individuals with PMS have impairments in social approach and engagement behaviors, despite struggling with directing attention more than children with iASD [40]. Thus, while ASD is a clinical syndrome comprising a combination of features where social deficits are prominent, there are few studies that explore how deficits in specific social behaviors present in PMS.

It has been suggested that the behavioral manifestations of social dysfunction in ASD are the consequence of atypical attentional processing [12]. Typically developing infants display a sensitivity to social cues in their environment [49], and an attentional predisposition toward social stimuli is necessary for effective communication and language acquisition [13]. Individuals with ASD, however, often fail to orient to social stimuli [15] and also show an absence of preferential attention [24] and impaired recognition [7] particular to social input. In a visual paired-comparison task (VPC) that tested visual memory by pairing an unfamiliar stimulus with a familiar one, toddlers with ASD showed differential eye gaze patterns and reduced recognition memory for social stimuli compared to typically developing (TD) controls [9]. Here, preferential looking toward the novel stimuli infers recognition memory of the familiar stimulus [19]. Orienting to non-social stimuli, on the other hand, appears intact in ASD [15]. Together, these results reveal a reliable pattern of atypical attentional and memory processes for social information in ASD. However, it is unknown whether a similar pattern of visual attention and memory impairments specific to social stimuli is also present in PMS and how other clinical symptoms of PMS impact the presentation. The paucity of experimental data on PMS is in part attributable to the rare nature of the syndrome.

Moderate to profound ID affects approximately 75% of individuals with PMS [48]. Deficits in short-term memory are characteristic of a person with ID [1] and recognition memory indexed by visual attention preference scores measured with the VPC task in infants has shown a relationship to performance on standardized intelligence test later in life [45, 51]. Thus, clarifying whether potential deficits in visual attention and memory relate to the ASD phenotype or the presence of comorbid ID in a PMS sample is an important point of consideration.

The goal of the present study was to examine processing of social stimuli in PMS, in comparison to iASD and TD controls, using a visual paired-comparison task to test for syndrome-specific attentional and memory deficits of social information. We hypothesized, consistent with previous literature, that social attention and memory in iASD groups are atypical regardless of cognitive functioning, such that novelty preference for social stimuli would be reduced in comparison to non-social stimuli. We further expected that, relative to iASD groups, individuals with PMS would have a global deficit in memory and attention that was less specific to social recognition memory and more consistent with broad attentional and intellectual challenges. Finally, we predicted that individuals with PMS with comorbid ASD diagnoses would have social attention and memory profiles more similar to iASD than would individuals with PMS without an ASD diagnosis.

## Method

### Participants

Eye tracking data were collected from 85 5- to 18-year-old participants with iASD (*n* = 38), PMS (*n* = 22), and TD controls (*n* = 28). Following data cleaning (see below), the final sample consisted of 38 participants with iASD, 22 with PMS, and 26 TD controls. Legal guardians gave consent, and assent was obtained from participants when appropriate. Procedures were approved by the Program for the Protection of Human Subjects at the Icahn School of Medicine at Mount Sinai.

For the iASD and PMS groups, clinical diagnosis was confirmed with the Autism Diagnostic Observation Schedule (ADOS-2 [29];) and the Autism Diagnostic Interview-Revised (ADI-R [30];), followed by clinical consensus among licensed clinical psychologists and psychiatrists. All individuals in the iASD group met DSM-5 [2] criteria for ASD; in the PMS group, 14 participants (64%) met criteria for ASD. For the PMS group, chromosomal microarray or targeted sequencing was conducted to verify genetic diagnosis. Pathogenic deletion or mutation of the *SHANK3* gene was necessary and sufficient for a PMS diagnosis. All individuals in the idiopathic ASD group had a negative array resulting from genetic testing. The PMS group was divided into participants with a co-morbid ASD diagnosis (PMS^+^; *n* = 14; females: 8 (57.1%)) and those without an ASD diagnosis (PMS^−^; *n* = 8; females: 4 (50.0%)).

Participants were administered standardized measures of intellectual or developmental functioning. Depending on age and functioning level, participants received one of the following tests: the Stanford-Binet Intelligence Scales (SB-5 [41];), the Wechsler Intelligence Scale for Children (WISC-V [57];), the Wechsler Abbreviated Scale of Intelligence (WASI-II [56];), the Differential Ability Scales (DAS, DAS-II [18];), or the Mullen Scales of Early Learning [33]. Developmental quotients [DQ: (mental age/chronological age * 100)] scores were computed from subtest-level age equivalent scores in order to combine data across the different IQ tests administered. The iASD group was divided by IQ to form a subgroup with intellectual disability to match with the PMS group, scoring DQ < 70 (iASD-lo; *n* = 7) and a second subgroup with DQ > 70 (iASD-hi, *n* = 31). This resulted in five groups: PMS^+^, PMS^−^, iASD-lo, iASD-hi, and TD controls. Table 1 shows a summary of assessment scores.

**Table 1:** Participant demographics

	Group means (mean, SD)					Statistics	
	iASD-hi (*n* = 31)	iASD-lo (*n* = 7)	PMS^+^ (*n* = 14)	PMS^−^ (*n* = 8)	TD control (*n* = 26)	*F* or *χ*	*p*
Age	9.65 (3.31)	7.78 (2.73)	9.86 (4.17)	9.23 (3.41)	9.83 (3.72)	0.55	0.70
DQ	106.68 (24.24)	45.50 (21.08)	17.29 (12.16)	35.82 (19.83)	153.80 (56.25)	49.67	< 0.001
ADOS^a^	7.66 (1.54)	6.80 (2.17)	7.29 (1.54)	6.00 (2.20)	—	1.61	0.22
ADI-R (A)^b^	18.33 (5.69)	17.00 (5.15)	22.08 (6.10)	13.00 (9.20)	—	2.78	0.071
ADI-R (B verbal)^b^	15.50 (4.99)	13.20 (2.78)	13.77 (3.77)	10.43 (4.08)	—	3.31	0.032
ADI-R (B non-verbal)^b^	8.21 (4.44)	8.40 (3.85)	12.69 (4.13)	6.43 (3.60)	—	5.09	0.006
ADI-R (C)^b^	7.17 (2.53)	6.60 (2.51)	5.70 (2.63)	4.00 (2.77)	—	2.93	0.054
ADI-R (D)^b^	3.75 (1.29)	4.20 (1.79)	4.62 (0.77)	3.86 (1.22)		1.23	0.34
Sex (% female)	7 (22.6%)	2 (28.6%)	8 (57.1%)	4 (50.0%)	17 (65.3%)	12.36	0.015
# Soc trials	4.93 (0.04)	4.86 (0.09)	4.83 (0.06)	4.83 (0.08)	4.99 (0.05)	0.65	0.63
# Non-soc trials	4.0 (0.03)	3.91 (0.7)	3.86 (0.05)	3.83 (0.06)	3.95 (0.04)		
Race (*n* = Asian)	3	0	1	0	0	—	—
Race (*n* = Black or African American)	5	2	0	0	1	—	—
Race (*n* = White)	13	2	11	8	20	—	—
Race (*n* = More than one)	6	1	2	0	3	—	—
Race (*n* = Unknown)	4	2	0	0	2	—	—
Ethnicity (*n* = Not Hispanic)	16	4	13	7	20	—	—
Ethnicity (*n* = Unknown)	8	2	0	0	4	—	—

^a^Score reported are calibrated severity scores with missing values for 4 participants (7%) (iASD: *n* = 4)

^b^ADI-R scores were missing for 11 participants (19%) (iASD, *n* = 9; PMS, *n* = 2)

### Apparatus and testing procedure

An EyeLink 1000 plus eye-tracker in head-free mode with a 17-inch LCD monitor and 1280 × 1024 pixel at 32 bits per color display, with a refresh rate of 60 Hz was used for data collection. Data were acquired at 500 Hz with a 5- or 13-point calibration used on each participant before the start of the task.

Testing was performed in a dark, quiet room. Participants were positioned at a small table in either a chair or a booster seat roughly 50 cm from the eye tracker and monitor. Ambient sound and light were minimized in order to reduce distraction and tracking interference. The experimenter would give generic verbal instructions: “Look at the images you see on the screen. Between each set of images, look at a dot in the center of the screen.” Importantly, comprehension of these instructions was non-critical; participants were allowed to passively view on-screen images, and the trials were presented only when participants were fixating the screen (see below). In this way, the task was accessible to individuals with a wide range of cognitive and language abilities.

### Stimuli and experimental procedure

**Fig. 1:**
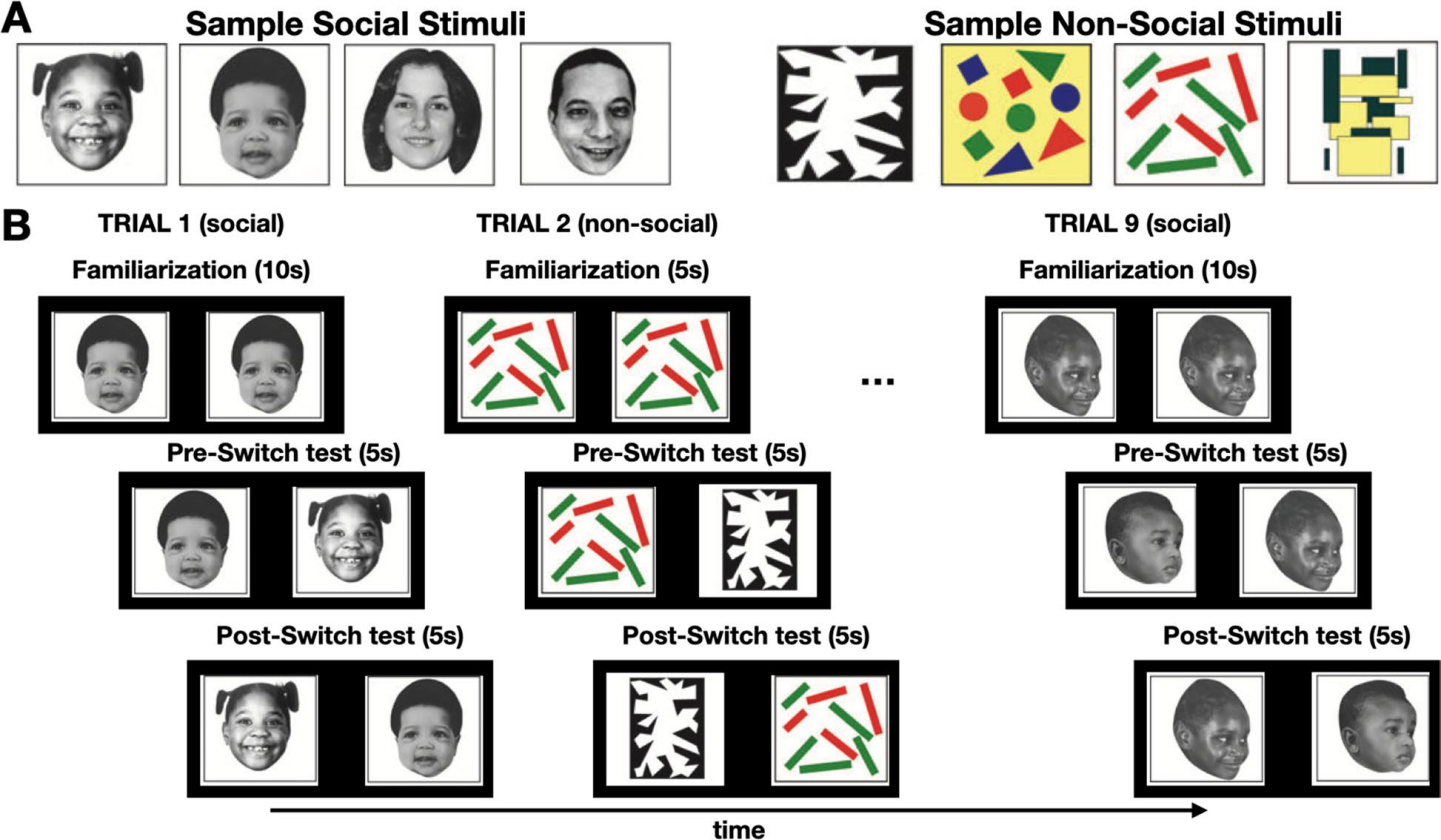
**A** Sample of social (left) and non-social (right) visual stimuli and (**B**) trial schematic of visual paired-comparison task. Familiarization was followed by a pre-switch test, which featured a novel image, and finally post-switch test when the novel and familiar images swapped sides. The task was comprised of 9 trials that alternated between social and non-social trial types

The visual stimuli, from Rose et al. [42], comprised of achromatic faces (social) and multicolored abstract patterns (non-social) (see Fig. 1A), on a black background. At a viewing distance of 50 cm, stimuli subtended a 14.2° × 10.2° visual angle and the inner edges of the two images subtended 8.28° of visual angle. The task was presented using the EyeLink Experiment Builder software application.

This study used the well-established visual paired-comparison paradigm [20, 42]. Trials began with a *familiarization* period. During this phase, identical images were presented in pairs, one image on the left and one on the right of the vertical meridian. As per Rose et al. [42], viewing times were 10 s (social images) or 5 s (non-social images), excluding the time when the participant’s eye gaze left the display monitor. Prior research had found that these minimal familiarization periods were needed to achieve habituation effects [43]. Next, during the *test* period, the image displayed during familiarization was paired with a novel image for 5 s (pre-switch), after which the two images switched sides and were displayed for an additional 5 s (post-switch) (Fig. 1B). The familiarization and test periods were not blocked; rather, each trial began with a familiarization period that preceded the test period. Each trial featured a unique set of stimuli and the novel stimulus was never used as a stimulus during any of the familiarization periods. The location of the novel stimulus was balanced across stimulus types and test periods. As per Rose et al. [42], participants viewed nine trials in total: five social and four non-social. Order of social and non-social trials was fixed and alternated between the two trial types. A flashing target and a loud “spaceship” noise were shown for a few seconds between each trial to ensure participants returned to a central, neutral fixation point. The experimenter would visually confirm the position of the participant’s eye gaze before manually initiating the start of each trial.

### Data analysis

Areas of interest (AOI) were defined as the rectangular area encompassing the image (AOI size, 14.2° × 10.2° of visual angle). Trials were excluded from analysis if *familiarization*/*test* period contained valid eye gaze for less than 25% of the total time within the AOI indicating that they were attending to the images less than 25% of the total time (social familiarization, 2.5 s; non-social familiarization, 1.25 s; test, 1.25 s). This threshold has been used previously in eye tracking studies [21]. Participants were excluded from analysis if they had less than two trials per condition. The trial minimum requirement used was based on infant literature where single trials are typical and sufficient for estimating looking behavior [10, 23]. Two TD participant met the exclusion criteria threshold (mean age, 7.11 (3.78), number female, 1 (50%), DQ, 133.23 (54.78)). There were no significant differences in the number of included trials among familiarization/test phases (*F*_2,162_ = 3.03, *p* = 0.051, *η*_p_^2^ = 0.036; familiarization, 4.44 ± 0.025; pre-switch test, 4.37 ± 0.036; post-switch test, 4.36 ± 0.31), or by group (*F*_4,81_ = 1.53, *p* = 0.20, *η*_p_^2^ = 0.07; TD, 4.47 ± 0.037; iASD-hi, 4.44 ± 0.34; iASD-lo, 4.38 ± 0.072; PMS^−^, 4.33 ± 0.67; PMS^+^, 4.35 ± 0.051). There was a significant difference in the number of remaining trials by stimulus type (*F*_1,81_ = 1721.66, *p* < 0.001, *η*_p_^2^ = 0.96; social, 4.89 ± 0.031; non-social, 3.9 ± 0.023) likely driven by the different number of possible trials for the two conditions (5 social and 4 non-social). None of the interaction reached statistical significance (period×group: *F*_8,162_ = 0.86, *p* = 0.57, *η*_p_^2^ = 0.041; stimulus type×group: *F*_1,81_ = 0.65, *p* = 0.63, *η*_p_^2^ = 0.031; period×stimulus type: *F*_2,162_ = 0.61, *p* = 0.54, *η*_p_^2^ = 0.008; period×stimulus type×group: *F*_2,162_ = 0.53, *p* = 0.83, *η*_p_^2^ = 0.026).

In *familiarization*, total visit duration (TVD) in AOIs, corrected for total display time difference of stimulus type, was calculated for both the left and right image, then summed to determine the proportion of total image exploration time. The data were subjected to a 2 × 5 mixed analysis of variance (ANOVA) with stimulus type (social, non-social) as the within-subjects factor and diagnosis (iASD-hi, iASD-lo, PMS^+^, PMS^−^, TD controls) as the between-subject factor. In addition, a rate of entering the AOI established the amount of switching back and forth between the two identical images and was analyzed with 2 × 2 × 5 mixed ANOVA with stimulus type, location (left, right), and diagnosis as levels of the independent variable (IV). The rate was calculated using the number of entries into the AOI divided by the exposure time (social, 10 s; non-social, 5 s).

During *test*, total visit duration for each image (novel and familiar) was calculated separately for the initial 5 s presentation (pre-switch) and after the images switched sides (post-switch). A preference score was calculated as the average time spent on the AOI for the familiar image subtracted from the time spent on the novel image divided by the sum of time spent on either image: (*novel* − *familiar*)/(*novel* + *familiar*). A score significantly different from zero in either direction (positive for novelty or negative for familiarity) was indicative of recognition memory. Preference scores were subjected to a 2 × 5 repeated measures ANOVA with switch period (pre-, post-), and diagnosis as IV levels for each stimulus type. One sample *t* tests were conducted separately for each clinical group to analyze the existence of a preference (i.e., significant difference from zero/no preference, where equal time is spent on the familiar and novel images).

All post hoc group comparisons were subjected to Games-Howell corrections for multiple comparisons to account for unequal variance. All other tests were Bonferroni corrected with a two-tailed 0.05 significance criterion.

## Results

### Clinical and demographics measures

The final sample showed no significant difference among groups for age, *F*_4,52.11_ = 0.55, *p* = 0.70, *η*_p_^2^ = 0.025. There was a significant difference in DQ, *F*_4,29.716_ = 49.67, *p* < 0.001, *η*_p_^2^ = 0.72, with eleven participants missing DQ scores (iASD-hi, 1; iASD-lo, 0; PMS^+/−^, 0; TD, 10). A clinician estimate of DQ score > 70 based on clinical impressions and patient history was determined for the one iASD-hi participant without a formal IQ assessment available. The mean DQ score of the TD control group was higher than the iASD-hi, iASD-lo, PMS^+^, and PMS^−^ groups (all *ps* < 0.04). The iASD-hi group also had significantly higher DQ scores compared to the PMS groups (*p* < 0.001 Games-Howell). However, the iASD-lo group did not differ from either PMS^−^ (*p* = 0.89 Games-Howell) or PMS^+^ (*p* = 0.063 Games-Howell) groups, and the PMS groups did not differ from one another in DQ (*p* = 0.18 Games-Howell). A significant sex difference was also found among the groups, *χ* (4) = 12.36, *p* = 0.015; a post hoc test of the adjusted residuals tested for groups statistically different from the expected equal distribution of sex across groups (i.e., iASDs=PMSs=TD). Here, it was revealed that the iASD-hi group (*p* = 0.002) was significantly different from the expected (null hypothesis) value of no difference in sex count. Follow-up comparisons using a two-sample Kolmogorov-Smirnov test found the iASD-hi group had a greater proportion of males when compared with the TD control group (*p* = 0.011); this did not survive significance after correcting for multiple comparisons. Nevertheless, this difference is consistent with the male:female ratio in ASD [3], whereas males and females are equally affected with PMS [36, 54]. No other comparisons for sex ratio were found to be significant after correcting for multiple comparisons (iASD-hi/iASD-lo, *p* = 1.00; iASD-hi/PMS^−^, *p* = 1.00; iASD-hi/ PMS^+^, *p* = 0.96).

### Familiarization

**Fig. 2:**
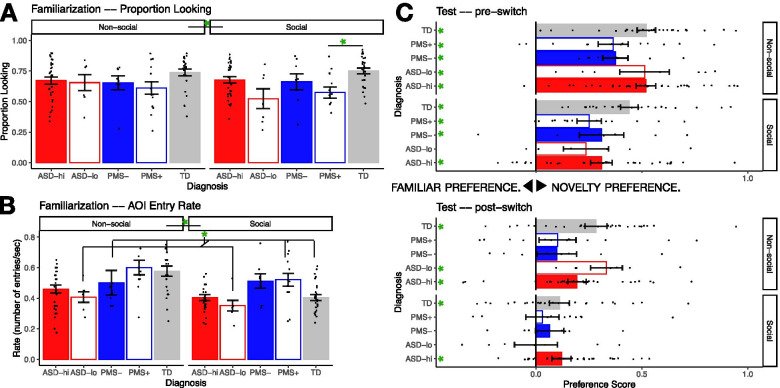
Familiarization results. **A** Proportion looking during social and non-social trial types for the iASD-hi (red, filled), iASD-lo (red, open), PMS^−^ (blue, filled) and PMS^+^ (blue, open), and TD controls (gray). **B** Average area of interest (AOI) image entry rate for social (right) and non-social (left) trial types averaged across location. The iASD-lo (red, open) had a lower AOI entry rate compared to the PMS^+^ (blue, open) and TD control group (gray). **C** Test results. top: pre-switch test period for social and non-social preference scores (red-filled, iASD-hi; red-open, iASD-lo; blue-filled, PMS^−^; blue-open, PMS^+^; gray, TD controls) and bottom: post-switch test period. Values above zero denote a novelty preference and values below zero a familiarity preference. During the pre-switch period, all groups demonstrated a novelty preference for non-social images. In the post-switch period, the PMS subgroups and iASD-lo did not exhibit an image preference for social stimuli. Error bars represent ± 1 SE. *p < 0.5, **p < 0.01

Attention to social and non-social stimuli was compared among groups during the *familiarization* period. There was a significant main effect of stimulus type (*F*_1,81_ = 4.16, *p* = 0.045, *η*_p_^2^ = 0.049) and diagnostic group (*F*_4,81_ = 3.13, *p* = 0.019, *η*_p_^2^ = 0.134), and a significant stimulus by diagnosis interaction (*F*_4,81_ = 2.87, *p* = 0.030, *η*_p_^2^ = 0.12) on measures of proportion looking time. Across all groups, participants tended to look longer at non-social stimuli (0.67 ± 0.021) than looking at the social stimulus (0.64 ± 0.02). Between groups, no comparisons survived corrections for multiple comparison (*p* > 0.05, Games-Howell). The significant interaction effect was parsed into two separate ANOVAs by stimulus type. There was a significant group difference for social stimuli (*F*_4,81_ = 4.80, *p* = 0.002, *η*_p_^2^ = 0.19), where the PMS+ group tended to look significantly less to the social images in comparison to the TD controls (*p* = 0.020). There were no significant group differences in looking behavior in the non-social images condition (*F*_4,81_ = 1.56, *p* = 0.19, *η*_p_^2^ = 0.072). These findings indicate engagement with social images was less than engagement with non-social stimuli across groups and that, for social images, the PMS+ group engaged less than TD controls (Fig. 2A).

To investigate differences in looking patterns, we analyzed the rate of saccade entries into the left and right images. We found a significant main effect for stimulus type (*F*_1,81_ = 18.23, *p* < 0.001, *η*_p_^2^ = 0.18). Across all groups, participants had a higher rate of AOI entries to non-social stimuli (0.51 ± 0.02) than social stimuli (0.44 ± 0.02). There was no significant main effect of image location, i.e., left (0.47 ± 0.02) versus right (0.48 ± 0.02) image, *F*_1,81_ = 2.27, *p* = 0.14, *η*_p_^2^ = 0.027. However, a statistically significant main effect of group (*F*_4,81_ = 3.66, *p* = 0.009, *η*_p_^2^ = 0.15) was detected wherein, overall, the iASD-lo group made less entries into the AOIs (0.34 ± 0.05) compared with the PMS^+^ (0.56 ± 0.03; *p* = 0.009, Games-Howell) and the TD control groups (0.49 ± 0.03; *p* = 0.043, Games-Howell). No other comparisons with the TD control group were found to be significant (TD/iASD-hi: *p* = 0.38; TD/PMS^−^ (0.51 ± 0.05): *p* = 0.99; TD/PMS^+^: *p* = 0.55). Comparisons with iASD-hi (0.43 ± 0.02) were neither significant (iASD-hi/iASD-lo, *p* = 0.54; iASD-hi/PMS^−^, *p* = 0.77; iASD-hi/PMS^+^, *p* = 0.06) nor were the other comparisons with iASD-lo and PMS^−^ (*p*=0.37) or PMS^−^ and PMS^+^ (*p* = 0.94).

A significant group×stimulus type interaction (*F*_1,80_ = 4.60, *p* = 0.002, *η*_p_^2^ = 0.19) was detected. Follow-up one sample *t* tests examining the AOI entry rate difference between non-social and social images (non-social—social) revealed that the TD control group showed the largest difference in AOI entry rate between social and non-social (mean difference, 0.17 ± 0.03; *p* < 0.001) stimuli favoring non-social images, followed by the iASD-hi group (0.06 ± 0.02; *p* = 0.006). The PMS groups showed neither significant difference between entry rate between stimulus types (PMS^−^, −0.01 ± 0.05; *p* = 0.84; PMS^+^, 0.08 ± 0.04; *p* = 0.77) nor did the iASD-lo group (0.06 ± 0.04; *p* = 0.26). There was no significant group×location interaction, *F*_4,81_ = 1.75, *p* = 0.15, *η*_p_^2^ = 0.1. No other interactions were significant (stimulus type×location: *F*_1,81_ = 0.29, *p* = 0.59, *η*_p_^2^ = 0.004; group×stimulus type×location: *F*_4,81_ = 0.71, *p* = 0.59, *η*_p_^2^ = 0.03) (Fig. 2B). In sum, engagement with stimuli differed among the groups in looking pattern by stimulus type in addition to overall time on screen.

### Test

The *test* period introduced a novel image, evaluating memory through preferential looking toward the newly presented social and non-social stimuli. Due to the significant differences of attentional engagement between stimulus type, analyses were performed by social and non-social conditions separately. In the social conditions, we found a significant main effect of test period (pre-/post-switch: *F*_1,81_ = 39.17, *p* < 0.001, *η*_p_^2^ = 0.33). Across groups, participants looked less at the novel image during the post-switch period (−0.066 ± 0.03) compared with the pre-switch test period (0.31 ± 0.3). There was no significant main effect of group (*F*_4,81_ = 1.50, *p* = 0.21, *η*_p_^2^ = 0.07). There were no significant interactions between test period×group: *F*_4,81_ = 0.80, *p* = 0.53, *η*_p_^2^ = 0.04).

An analysis of the non-social condition revealed similar results as for the social condition, namely, that there was a significant main effect of test period (*F*_4,81_ = 55.03, *p* < 0.001, *η*_p_^2^ = 0.41), where there was a stronger novelty preference pre-switch (0.46 ± 0.03) than post-switch (−0.20 ± 0.03). As for the social condition, in the non-social condition, there was no significant main effect of group (*F*_4,81_ = 22.17, *p* = 0.080, *η*_p_^2^ = 0.10) or an interaction effect (*F*_4,81_ = 0.62, *p* = 0.65, *η*_p_^2^ = 0.03). In aggregate, these results indicate similarity among groups in their condition-specific recognition memory.

Finally, to test for the presence of a novelty/familiarity preference for each group and stimulus type, we ran one-sample *t* test on each group for each condition and test period (Fig. 2C). It was found that the TD control group had a novelty preference in the pre-switch test for both stimulus types (non-social, 0.52 ± 0.04, *p* < 0.001; social, 0.44 ± 0.04, *p* < 0.001). During the post-switch period, TD controls also demonstrated a novelty preference for non-social stimuli (0.28 ± 0.05, *p* < 0.001) and social stimuli (0.11 ± 0.05, *p* = 0.026). Similar to the TD control group, the iASD-hi group showed a novelty preference pre-switch across conditions (non-social, 0.52 ± 0.05; *p* < 0.001; social, 0.31 ± 0.05, *p* < 0.001) and a novelty preference post-switch for non-social ( 0.19 ± 0.04; *p* < 0.001) and social stimuli (0.12 ± 0.04, *p* = 0.012). The iASD-lo group displayed a novelty preference during the pre-switch period for non-social (0.51 ± 0.12, *p* = 0.005) but not for social (0.23 ± 0.11, *p* = 0.069) stimuli and similarly during post-switch (non-social, 0.33 ± 0.1, *p* = 0.004; social, 0.0005 ± 0.10, *p* = 1.00). The PMS^−^ group had a novelty preference pre-switch (non-social, 0.38 ± 0.06, *p* < 0.001; social, 0.31 ± 0.11, *p* = 0.221) and no preference post-switch (non-social, 0.10 ± 0.09, *p* =0.32; social, 0.06 ± 0.07, *p* = 0.037) as did the PMS^+^ group (pre-switch: social, 0.25 ± 0.05, *p* = 0.001; non-social, 0.36 ± 0.07, *p* < 0.001; post-switch: social, 0.031 ± 0.08, *p* = 0.70, non-social, 0.10 ± 0.09, *p* = 0.27). This last finding suggests decay of memory in iASD-lo and PMS groups by the post-switch interval.

There were no significant correlations between sex and the eye tracking measures of proportion looking (social, *p* = 0.70; non-social, *p* = 0.50) or rate of entry (social left, *p* = 0.17; social right, *p* = 0.30; non-social left, *p* = 0.39; non-social right, *p* = 0.88) during *familiarization*, or preferences scores during *test* (pre-switch social: *p* = 0.96; pre-switch non-social, *p* = 0.15; post-switch social, *p* = 0.15; post-switch non-social, *p* = 0.15).

## Discussion

The present study in Phelan-McDermid syndrome examined two aspects of cognition that have been implicated in ASD: social attentional engagement and memory. This represents the first study to examine these constructs in PMS, one of the most common single-gene causes of ASD. We found that attentional engagement differed for social and non-social images for all groups and that individuals with PMS and a comorbid ASD diagnosis, but not those without comorbid ASD, had lower engagement compared to typically developing controls for social images specifically. In addition, the groups were dissociable in both their looking patterns and their preferential looking behavior toward novel versus familiar images. In particular, the iASD group with ID showed less active looking, as indexed by the entry rates, during stimulus familiarization compared to both PMS with an ASD diagnosis and typically developing controls. Interestingly, this pattern did not hold in participants with PMS without ASD who were similar in their cognitive functioning level. Finally, all groups showed a greater novelty preference for non-social (versus social) stimuli during the pre-switch test period. Results from the post-switch period revealed a novelty preference in both the autism group without ID and TD control group, while the iASD group with ID and both PMS groups demonstrated a lack of preference.

Our results in iASD are generally consistent with prior studies that report atypical processing of social stimuli in ASD. Namely, the lack of a novelty preference in the ASD with ID group on trials with social, but not non-social, images is in line with previous findings. A recent meta-analysis indicated that individuals with ASD are worse at remembering and discriminating facial identity [58] compared to TD controls, which is consistent with our findings. Some have attributed this domain-specific impairment for faces to reduced reward placed on social information [11]. Other research suggests that the complexity of faces presents a challenge for individuals with ASD in terms of discerning relevant and irrelevant information features, thereby affecting memory processes [53]. It is notable that within the iASD without ID group performance was comparable to typically developing controls. It cannot, however, be discounted that differences between social and non-social stimuli may have been in part been driven by the differences in the low-level features of the stimuli, such as color. While the stimuli were the same used in Rose et al. [42], the social stimuli were all gray scale images while the non-social images were in color. Past literature has found that color images are remembered more accurately than black and white images [59] and color images may have been interpreted as being more salient in attracting attention [52]. Future research would need to explore this further with stimuli better matched for low-level features.

During test (pre-switch), all groups except the iASD with ID group demonstrated a novelty preference across both stimulus types, indicating that participants were able to discriminate between the familiar and unfamiliar items. However, during post-switch, both PMS groups showed neither novelty nor familiarization, suggesting recognition memory had decayed over the 5 s pre-switch test period in these groups, whereas it remained intact for the TD control and both iASD groups. These results in PMS are consistent with studies of *Shank3*-deficient mice revealing specific deficits in the mediation of glutamatergic function [6], a neurotransmitter involved in learning and memory through long-term potentiation [32]. Individuals with ID consistently perform lower on assessments of memory ability than typically developing controls [47].

Interestingly, though the iASD group with ID showed a novelty-preference for non-social stimuli but not social, this condition-selective effect was not observed for ASD associated with PMS, wherein a novelty preference during initial presentation was observed for both social and non-social images. Participants with PMS and ASD looked more similar to participants with PMS without ASD, showing a novelty preference regardless of stimulus type. These findings suggest that the dissociation between social and non-social memory and novelty preference is less prominent in those with ASD caused by PMS relative to the typical profile observed in iASD more generally.

We found comparable viewing times among iASD, PMS without ASD, and TD control groups when participants were initially presented with identical image pairs during the familiarization phase. The lack of any differences suggests that each group had equitable cumulative looking times to become familiar with the images, which further suggests that differences in novelty preference were not due to differences in familiarity or attention. However, across groups, there were some notable differences in rates of looking away and returning to the image, which may measure the process of comparing the pair of images during initial exploration [44]. Those individuals with PMS and co-morbid ASD had the higher rates of looking between stimuli, whereas the iASD group with ID was significantly lower by comparison. The higher switching rate paired with the lower dwell time during familiarization for the PMS with ASD group suggest that the going back and forth between images impacted total viewing time, but did not have significant consequences on recognition memory during the initial test. Here again, results highlight a pattern of dissociable social impairments in ASD associated with PMS versus idiopathic ASD more broadly. In the iASD without ID and TD control groups, there was a tendency for more active looking at non-social versus social images. In the PMS without ASD group, overall rates of looking between images were comparable to the TD control group, but the difference in looking rates for social versus non-social stimuli observed in the other two groups was absent. These results could suggest that looking behaviors such as rate of entry, more than looking time, contribute to subsequent differences in memory and preference for social and non-social stimuli among groups. Previous research has suggested a high level of switching back and forth between items was correlated with better memory performance [26, 44]; why the PMS with ASD group shows intact rates of looking switches yet poorer memory by the post-test interval could be attributed to deficient long-term potentiation [6], in spite of intact initial encoding behavior and early (pre-switch) memory.

While the switch during the test period was introduced to control for side preferences, the difference in performance (a weaker novelty preference overall in the post-switch period) between the pre- and post-switch periods suggests that participants were not acting the same across the test periods. One possibility is that the abrupt change in images during the switch could be interpreted by some participants as a novelty of location, or memory was beginning to decay over time.

Study limitations are due primarily to factors inherent in studying rare disease populations. First, groups were not matched on DQ scores. ID is characteristic of the vast majority of individuals with PMS and we attempted to control for DQ differences by having an iASD group with ID that was comparable in DQ. The visual paired-comparison paradigm has been used successfully with infants as young as 5 months old [43], has minimal instructions, and is free-viewing in nature, which ought to make it suitable for all our groups, regardless of intellectual functioning. Furthermore, we found no group differences in looking time during image familiarization suggesting the PMS and iASD with ID groups were broadly attentive to the task, with their eye gaze on the display monitor. Despite these considerations, we cannot discount that memory performance in the PMS groups may be associated more with their intellectual disabilities than their ASD phenotype. These groups tended to have comparable performance despite differing on ASD status and performed more similar to the iASD group with ID, with whom they have ID in common. While this study is among the first to probe specific cognitive functioning in a task-based way, it will be important for future studies to IQ match and examine the drivers of this PMS phenotype, whether it be ID or ASD. In addition, including non-PMS ID groups without ASD for comparison would help clarify this issue. Second, our study included participants across a wide age range in order to recruit an adequate sample size given the rarity of PMS. However, the rarity of this disorder—and of empirical research describing core cognitive deficits—emphasizes its uniqueness and the importance of this work despite some sample limitations. Lastly, while stimuli differed across trials and between stimulus types, the images were taken from previously published studies and were calibrated for equal attractiveness within a trial as well as for habituation times reported for social and non-social images [42, 43]; however, it remains possible that differences between calibrated stimuli across social and non-social images and their presentation times could impact results in addition to the differences in the number of trials presented. In strictly following the experimental design of Rose et al. [42], we sought to replicate an established task with published results as closely as possible. As this study involved participants with a rare disorder who are difficult to recruit and test, grounding our experiment in previously published study was particularly important for providing background against which to contextualize our results.

## Conclusion

In summary, while we found no general attentional engagement impairments in iASD or PMS, there were signs of disorder and domain-specific memory impairments and looking patterns. We found that, overall, individuals with PMS show a novelty preference regardless of stimulus type, whereas individuals with iASD matched in cognitive functioning showed only a novelty preference for non-social stimuli. In addition, both looking behaviors and novelty preference differed between iASD and ASD in the context of PMS. These unique patterns of social attentional engagement and memory in PMS differentiate it from patterns typical of iASD and could both clarify underlying mechanistic alterations and inform treatment targets for PMS. In addition, searching for PMS-like attention and memory patterns could serve as a way to stratify individuals within heterogeneous iASD samples and potentially apply knowledge gained in PMS to these iASD subsets as well.

## Data Availability

Not applicable.
